# Effect of calcium supplementation on bone resorption in pregnancy and the early postpartum: a randomized controlled trial in Mexican Women

**DOI:** 10.1186/1475-2891-13-116

**Published:** 2014-12-16

**Authors:** Adrienne S Ettinger, Héctor Lamadrid-Figueroa, Adriana Mercado-García, Katarzyna Kordas, Richard J Wood, Karen E Peterson, Howard Hu, Mauricio Hernández-Avila, Martha M Téllez-Rojo

**Affiliations:** Center for Perinatal, Pediatric, and Environmental Epidemiology, Yale Schools of Public Health and Medicine, 1 Church Street 6th floor, New Haven, CT 06510 USA; National Institute of Public Health, Universidad 655 Colonia Santa María Ahuacatitlán, C.P. 62100, Cuernavaca, Morelos Mexico; School of Social and Community Medicine, University of Bristol, Bristol, England, UK; Department of Nutrition, University of Massachusetts at Amherst, Amherst, MA USA; Department of Environmental Health Sciences, Human Nutrition Program, University of Michigan School of Public Health, Ann Arbor, MI USA; Dalla Lana School of Public Health, University of Toronto, Toronto, ON Canada

**Keywords:** Bone-specific alkaline phosphatase, Calcium, Clinical trials, Lactation, Pregnancy, Quantitative ultrasound bone speed of sound, Urinary N-telopeptide of type I collagen

## Abstract

**Background:**

Calcium needs are physiologically upregulated during pregnancy and lactation to meet demands of the developing fetus and breastfeeding infant. Maternal calcium homeostasis is maintained by hormonal adaptive mechanisms, thus, the role of dietary calcium supplementation in altering maternal responses to fetal-infant demand for calcium is thought to be limited. However, increased calcium absorption is directly related to maternal calcium intake and dietary supplementation has been suggested to prevent transient bone loss associated with childbearing.

**Methods:**

In a double-blind, randomized placebo-controlled trial, we randomly assigned 670 women in their first trimester of pregnancy to 1,200 mg/day calcium (N = 334) or placebo (N = 336). Subjects were followed through 1-month postpartum and the effect on urinary cross-linked N-telopeptides (NTx) of type I collagen, a specific marker of bone resorption, was evaluated using an intent-to-treat analysis. Women with a baseline and at least one follow-up measurement (N = 563; 84%) were included. Subsequent analyses were conducted stratifying subjects by compliance assessed using pill counts. In random subsets of participants, bone-specific alkaline phosphatase (BAP) (N = 100) and quantitative ultrasound (QUS) (N = 290) were also measured.

**Results:**

Calcium was associated with an overall reduction of 15.8% in urinary NTx relative to placebo (p < 0.001). Among those who consumed ≥50%, ≥67%, and ≥75% of pills, respectively, the effect was associated with 17.3%, 21.3%, and 22.1% reductions in bone resorption (all p < 0.001). There was no significant effect of calcium on bone formation measured by BAP. However, by 1-month postpartum, those in the calcium group had significantly lower NTx/BAP ratios than those in the placebo group (p = 0.04) indicating a net reduction in bone loss in the supplement group by the end of follow-up. Among subjects who consumed ≥50% and ≥75% of pills, respectively, calcium was also associated with an increase of 26.3 m/s (p = 0.03) and 59.0 m/s (p = 0.009) in radial SOS relative to placebo by 1-month postpartum.

**Conclusions:**

Calcium administered during pregnancy and the early postpartum period, to women with intakes around adequacy, was associated with reduced bone resorption and, thus, may constitute a practical intervention to prevent transient skeletal loss associated with childbearing.

**Trial registration:**

ClinicalTrials.gov Identifier NCT00558623

## Background

Calcium needs are physiologically-upregulated during pregnancy and lactation to meet the demands of the developing fetus and breastfeeding infant for skeletal mineralization and growth [1, 2]. Maternal calcium homeostasis is maintained by hormonal adaptive mechanisms that control intestinal calcium absorption, renal calcium excretion, and mobilization of skeletal mineral stores [3, 4]. The role of dietary calcium supplementation in altering maternal responses to fetal-infant demand for calcium is thought to be limited; however, increased calcium absorption is directly related to maternal calcium intake [5, 6].

Pregnancy- and lactation-associated bone loss has also been demonstrated through decreases in bone mineral density (BMD). An estimated five percent or more of total maternal bone mass may be mobilized [7, 8], although, this bone loss is reversible with levels rebounding to pre-pregnancy levels after cessation of lactation [9]. There is clear histological and biochemical evidence that the maternal skeleton undergoes increased bone resorption during pregnancy [10, 11].

Biochemical markers of bone resorption (osteoclast activity) and bone formation (osteoblast activity) have been found change drastically during pregnancy suggesting a physiological state of high bone turnover [12]. These markers of bone turnover may identify changes in bone remodeling and microarchitecture within a relatively short time interval (several days to months) before changes in BMD can be detected [13] and, thus, may provide insights into mechanisms of bone loss [14]. The long-term effects of these transient changes in maternal bone on child bone health are not fully understood [15], but new data indicate that maternal dietary deficiency during pregnancy may be associated with lower peak bone mass in offspring [16, 17].

It is recommended that U.S. pregnant and breastfeeding women over the age of 18 years consume at least 1,000 mg calcium per day [18], but these recommendations are based largely on studies in non-pregnant adults [2]. High dietary calcium intake has been shown to decrease bone mobilization during pregnancy [19, 20] suggesting that dietary calcium supplementation may be an effective means to prevent maternal bone loss. A number of studies have demonstrated an association with calcium supplementation and changes in bone turnover in non-pregnant adults [21], but data on the effects among pregnant women are scarce and there have been relatively few controlled supplementation trials that have studied the relationships directly [22]. The previously published trials of calcium supplementation and bone turnover in pregnant women [23–25] have been limited by their sample sizes and varying study designs making inferences from their results difficult. In addition, the trials in Gambia and China studied populations with low habitual dietary calcium intakes which limit their generalizability to populations with intakes approaching adequacy (such as the general U.S. population).

The objective of the present study was to evaluate the effect of dietary calcium supplementation on bone turnover during pregnancy and the early postpartum period using a double-blind, randomized placebo-controlled trial design. The hypothesis was that a daily supplement of 1,200 mg calcium carbonate would decrease bone resorption over the course of pregnancy among a relatively large sample of women with near adequate dietary calcium intakes.

## Methods

### Study population and design

**Figure 1:**
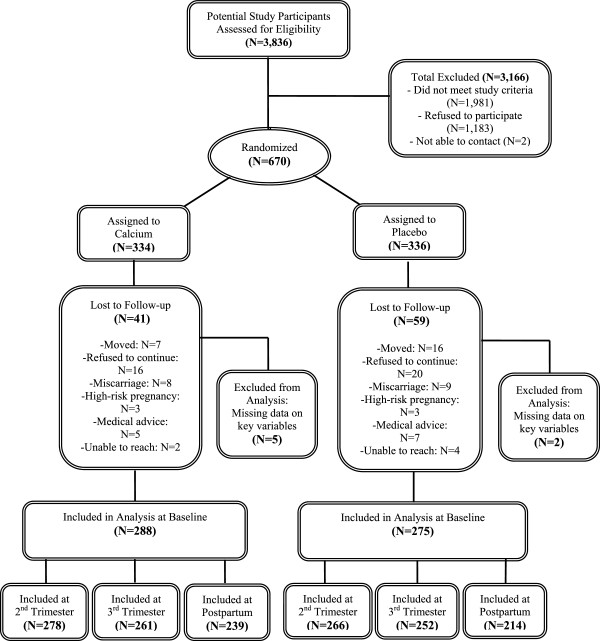
**Study sample profile.**

First trimester pregnant women were enrolled from January 1, 2001 to April 26, 2004 at Mexican Social Security Institute prenatal clinics which serve a low-to-moderate income population in Mexico City. In brief, a total of 3,836 women were assessed for eligibility, of whom 1,981 did not meet study eligibility criteria (pregnancy of no more than 14 weeks gestation; not a high-risk pregnancy; plans to reside in Mexico City for study period; and no other reasons for exclusion) or were not able to be reached for contact (N = 2). When pregnant women were screened for initial recruitment, they were excluded if they exhibited any of the following conditions: any factor that could interfere with maternal calcium metabolism, intention not to breastfeed, preeclampsia, kidney or cardiac diseases, gestational diabetes, history of urinary infections, family or personal history of kidney stone formation, seizure disorder requiring daily medications, or ingestion of corticosteroids. Of the remaining 1,853 eligible women, 670 (36%) agreed to participate, signed informed consent, and were randomly assigned to receive a daily supplement of 1,200 mg calcium carbonate (two-600 mg tablets (Lederle, Inc.); N = 334) or placebo (N = 336)(Figure 1). Neither participants nor study personnel were aware of treatment group assignments and placebo tablets were formulated to be indistinguishable from the active treatment tablets.

Calcium carbonate is ~40% elemental calcium by weight [26]; therefore, for 1,200 mg calcium carbonate, the elemental calcium equivalent is: 480 mg. All treatment and control subjects were provided with a daily supplement of 30 mg iron (Fe) sulfate from study entry through 12 months postpartum since prenatal vitamins were not included in the standard of care. Women were instructed to consume Fe supplements at the mid-day “comida” (main meal) to decrease side effects that may accompany Fe ingestion [27]. Supplement levels were selected to meet two criteria: ensured adequacy and safety of total dietary intake. Doses consistent with the AI for calcium [28] and the Recommended Dietary Allowance (RDA) for Fe [29] to ensure normal physiologic requirements for pregnancy and lactation [30] would be met among women in the lower quartile of intake in our study population. Calcium was suggested to be consumed at bedtime, rather than in the morning, due to recent evidence (at the time of study planning) of potentially greater effects on bone turnover which was shown to be greater during the night than daytime hours [31]. Given potential problems with compliance, a split-dose regimen is not usually suggested for long-term supplementation trials as simplified drug-dosing regimens have been shown to improve adherence to therapy [32].

Participants were assessed at four time points: baseline (1st trimester) prior to initiation of treatment, and after having consumed calcium or placebo at 6 (2nd trimester) and 8 (3rd trimester) months of gestation and 1-month postpartum. Immediately following the baseline assessment, women were instructed to consume tablets daily at bedtime and compliance was assessed by pill count at each follow-up visit. Women who had a baseline and at least one follow-up measurement (calcium, N = 288; placebo, N = 275) were defined as having completed follow-up and included in this analysis (N = 563; 84%). The reasons for loss-to-follow up and the final numbers of women included at each stage of the analysis are detailed in Figure 1.

The research protocol was approved by the Human Subjects Committees of the Mexican National Institute of Public Health, the Mexican Social Security Institute, and participating institutions and has complied with all federal guidelines governing the use of human subjects. All participants received a detailed explanation of the study intent and procedures prior to signing the informed consent.

### Markers of bone turnover

Urinary excretion of cross-linked N-telopeptides (NTx) of type I collagen was measured in urine from second-morning void collected by participants prior to each visit. NTx is a specific marker of osteoclast activity (bone resorption) that has been shown to be stable and resistant to degradation in stored samples [33]. Samples were analyzed with a commercially available competitive-inhibition enzyme-linked immunosorbent assay (Osteomark; Ostex International, Seattle, Washington). NTx concentrations were controlled for urine dilution using creatinine concentration and expressed as nanomoles of bone collagen equivalents (BCE) per millimole of creatinine (nM BCE/mM creatinine). The intra-assay CV was 8.9% (at 406 nM BCE) and 8.7% (at 1563 nM BCE); the inter-assay CV was 8.6% (at 427 nM BCE) and 5.6% (at 1513 nM BCE).

Bone-specific alkaline phosphatase (BAP) was measured in plasma stored at −70°C from a subset of participants (N = 100) using the Ostase® BAP immunoenzymetric assay (Immunodiagnostic Systems Inc., Fountain Hills, AZ). BAP levels reflect the metabolic status of osteoblasts and, thus, serve as an indicator of bone formation [34, 35].

### Bone ultrasound measurement

Bone speed of sound (SOS, in meters per second) was measured at the distal radius using quantitative ultrasound (QUS) (Sunlight Omnisense 7000, Zicon Ltd. Petah-Tikva, Israel) in a random subset of participants (N = 290). Dual-energy x-ray absorptiometry (DXA) is the gold standard for measuring BMD [36], however, due to the potential for ionizing radiation exposure to the fetus, its use during pregnancy is inadvisable and specifically prohibited by Mexican law. QUS allows for an inexpensive, convenient, and radiation-free method by which to assess bone quality during pregnancy and several previous epidemiologic studies have used quantitative ultrasound to assess bone changes over the course of pregnancy [37–39].

### Dietary intake

Daily intakes of calcium and total energy were assessed at each visit using a semi-quantitative food frequency questionnaire designed to estimate usual dietary intake over the prior month. The questionnaire was modified and validated among women living in Mexico City [40] and included questions specific to pregnancy such as any additional use of dietary supplements.

### Statistical analysis

To assess whether randomization was successful in achieving comparability, baseline characteristics were compared between the calcium and placebo groups using the Wilcoxon rank-sum (Mann–Whitney U) two-sample test of equality. A similar comparison was performed between those who were included in the analyses and those who were lost to follow-up in order to assess whether selective attrition occurred. All tests of statistical significance were two-sided.

The effect of the calcium supplement on bone resorption was evaluated using an intent-to-treat strategy. A first approach was to conduct a comparison of the log-transformed NTx concentrations between treatment groups at each follow-up stage, both unadjusted (t-test) and adjusting for covariates (linear regression). A second approach was fitting a by mixed-effects regression model with a random intercept for each subject in order to adjust for imbalances at baseline and to gain precision in treatment effect estimates by including covariates. Mixed-effects models take into account the correlation between repeated measures on subjects over time. In addition, as mixed models are flexible with respect to incomplete data, all subjects with at least one follow-up measurement were included to increase the study’s power. The outcome variable was natural log-transformed NTx in the 2^nd^ and 3^rd^ trimesters and 1-month postpartum. Models included the following baseline variables: treatment assignment (calcium vs. placebo), age (years), primigravidity (yes/no), NTx (nM BCE/mM creatinine), daily calcium (g/day) and energy intake (kcal/day), and time. We fitted a model including time*treatment interactions to test for heterogeneity of treatment effects at different timepoints. To assess if breastfeeding at 1-month postpartum modified the effect of the supplement, a cross-sectional model with an interaction term between lactation (0,1 variable that defines whether the woman was lactating at the time of the 1 month postpartum visit) and supplement group was also fitted.

A secondary strategy was to estimate the efficacy of the supplement by performing a dose–response analysis to further assess the effect of the supplement by estimated compliance. Compliance was analyzed as the proportion of the expected number of pills taken by subjects between consecutive visits and then categorized into three groups: ≥50% of pills consumed, ≥67% of pills consumed, and ≥75% of pills consumed.

We also fitted a model with the NTx/BAP ratio as the outcome variable, in the subset with both measures available (N = 100 subjects, 270 observations), to observe if the relative levels of bone resorption-to-bone formation changed over the course of the pregnancy and to evaluate if this change was different between treatment groups. All statistical analyses were performed using STATA for Windows, version 12.0 (StataCorp LP, College Station, Texas).

## Results

A total of 670 eligible women were randomized to receive calcium supplementation (N = 334) or placebo (N = 336) (Figure 1). Baseline characteristics were similar for the calcium and placebo groups with the exception of maternal age which was one year higher on average in controls (26.9 years) than in the supplement group (25.9 years; p = 0.02) (Table 1). Approximately 35.4% of women were primigravid and there were no significant differences by treatment. Dietary calcium intake, also not significantly different between treatment groups, was about 1,100 milligrams per day on average. Geometric mean (and geometric standard deviation (GSD)) pre-treatment NTx levels were 62.3 (1.7) and 62.9 (1.7) nM BCE/mM creatinine for the calcium and placebo groups, respectively (p = 0.73).

**Table 1:** **Baseline characteristics of subjects by treatment assignment and follow-up status**

	Treatment assignment		Follow-up status	
	Calcium	Placebo	Included ^a^	Not included
	(N = 334)	(N = 336)	(N = 563)	(N = 107)
Variable	Mean (SD)	Mean (SD)	Mean (SD)	Mean (SD)
Age (years)	26.9 (5.6)	25.9 (5.3)^b^	26.5 (5.5)	26.2 (5.3)
Education (years)	10.8 (2.9)	11.0 (3.2)	10.9 (3.1)	10.6 (2.9)
Number of pregnancies	2.0 (1.0)	2.1 (1.1)	2.1 (1.0)	2.0 (0.9)
Number of children	0.8 (0.8)	0.8 (0.9)	0.8 (0.9)	0.7 (0.7)
Number of months previous breastfeeding (cumulative lifetime)	5.6 (8.9)	6.8 (9.0)	6.4 (9.2)	5.1 (7.2)
BMI (kg/m^2^)	25.9 (4.1)	25.8 (3.7)	25.9 (3.9)	25.9 (3.9)
Energy intake (kcal/day)	1888 (592)	1862 (637)	1860 (613)	1951 (619)
Calcium intake (mg/day)	1108 (492)	1083 (532)	1096 (515)	1091 (497)
Hematocrit (%)	39.1 (3.3)	39.1 (3.0)	39.1 (3.2)	39.1 (2.7)
NTx (nM BCE/mM creatinine)^c^	62.3 (1.7)	62.9 (1.7)	62.9 (1.7)	52.2 (1.7)

^a^Defined as having at least one visit completed after baseline and included in final model.

^b^p < 0.05 Wilcoxon rank-sum (Mann–Whitney U) two sample test of equality of distributions.

^c^Geometric mean and GSD; n = 291 treated, n = 285 with placebo.

A total of 563 women (84%) had at least one follow-up assessment and were included in the analyses. Comparing those included in the analysis (placebo N = 275; calcium N = 288) to those who were not included (placebo N = 61; calcium N = 46) revealed no significant differences by treatment assignment suggesting that those women who remained in the study were not systematically different than those who did not complete follow-up. Overall, the proportion of lactating women at 1-month postpartum was 89.6% and there were no differences by treatment group (calcium, 89.9% vs. placebo, 89.3%; p = 0.8).

In the unadjusted intent-to-treat analysis, calcium was associated with average reductions of 15.1, 16.4, and 20.2% in NTx concentrations in the 2^nd^ and 3^rd^ trimesters, and 1 month post-partum respectively (all p ≤ 0.001). The corresponding visit-specific covariate-adjusted reduction estimates were 13.8, 15.6 and 19.2% (all p ≤ 0.001) (Table 2). The overall covariate-adjusted average reduction in NTx concentrations relative to placebo was 15.8% (p < 0.001).

**Table 2:** **Effect of calcium supplementation on NTX (Log-transformed) (N = 563)**

	Unadjusted			Adjusted ^a^		
	N	%∆ ^b^	p-value	N	%∆ ^b^	p-value
Study visit						
2^nd^ trimester	548	15.1	0.001	544	13.8	0.001
3^rd^ trimester	517	16.4	<0.001	513	15.6	<0.001
1-month postpartum	456	20.2	<0.001	453	19.2	<0.001
Average	567	16.8	<0.001	563	15.8	<0.001

^a^Adjusted for baseline: age, primigravidity, NTx, and dietary calcium and total energy intakes.

^b^Percent reduction: 1- e^β^.

**Figure 2:**
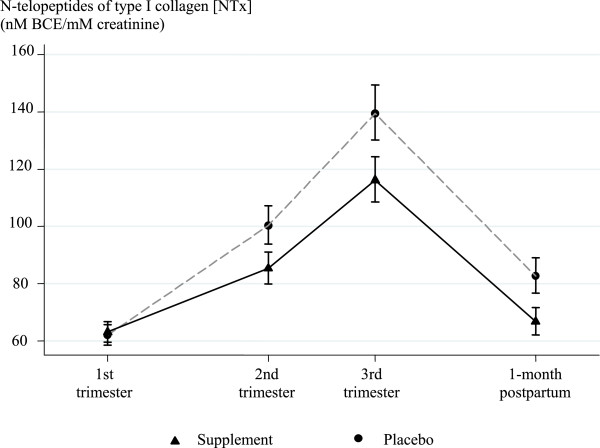
**Effect of calcium supplementation on urinary N-telopeptides of type I collagen [NTx] (nM BCE/mM creatinine) at each trimester during pregnancy and at 1-month postpartum (Intent-to-Treat Analysis, N = 563); adjusting for baseline variables: NTx, age, primigravidity, dietary calcium and daily energy intake.**

Results of the mixed effects regression model with treatment-by-time interactions showed a significantly different effect of the calcium supplementation on bone resorption at each study assessment when compared to baseline difference between treatment groups. The reduction was more evident at 1-month postpartum than in the 2nd and 3rd trimesters, but these reductions were significant for each of the three assessments: 2nd trimester (−13.7% reduction, p = 0.002); 3rd trimester (−15.6% reduction, p = 0.001); and 1-month postpartum: (−18.6% reduction, p < 0.001) (Figure 2).

Since response to treatment could depend on baseline dietary calcium intake, we tested a dietary calcium-by-treatment group interaction. There was no significant interaction between dietary calcium intake (either as a continuous variable or as quartiles) and supplement group at baseline. However, when examining lactation status, there was no effect of supplement in the non-lactating women (p = 0.57) compared to a 23% reduction in lactating women (p < 0.0001), indicating that lactation is an effect modifier for the effect of calcium supplementation on bone resorption.

When the effect of calcium supplementation was assessed for women “as treated” (N = 563) using models stratified by compliance (Table 3), we saw a dose–response effect of calcium on NTx concentration. Among those women who consumed ≥50% of pills, calcium was associated, on average, with a 17.3% reduction in NTx in comparison to placebo (p < 0.001). This increased to 21.3% (p < 0.001) and 22.1% (p < 0.001) for those who consumed ≥67% of pills and ≥75% of pills.

**Table 3:** **Effect of calcium supplementation** ^**a**^ **on NTx by treatment compliance** ^**b**^

		Average (Overall)		2nd trimester		3rd trimester		1-month postpartum	
Compliance	N (Obs)	%∆ ^c^	p-value	%∆ ^c^	p-value	%∆ ^c^	p-value	%∆ ^c^	p-value
ALL	563 (1510)	15.8	<0.001	13.7	0.002	15.6	0.001	18.6	<0.001
<50%	161^d^ (270)	11.2	0.110	10.9	0.256	10.7	0.383	12.6	0.361
≥50%	505^d^ (1240)	17.3	<0.001	14.9	0.003	15.6	0.002	19.2	<0.001
≥67%	378 (790)	21.3	<0.001	19.0	0.005	19.2	0.001	23.0	<0.001
≥75%	267 (423)	22.1	<0.001	25.0	0.171	19.0	0.006	21.9	0.002

^a^Adjusted for baseline: age, primigravidity, NTx, and dietary calcium and total energy intakes.

^b^Compliance assessed by pill count at each visit and analyzed as proportion of expected pills used between consecutive visits.

^c^Percent reduction: 1-e^β^.

^d^Numbers of subjects do not add to 563 because subjects may appear in more than one stratum due to time-varying nature of compliance.

The subset of women with serum BAP measurements (N = 100) were not significantly different than those who did not have the measurements available (N = 463) except for years in school (those with BAP had 0.7 more years, on average, p = 0.04) and hematocrit (those with BAP 0.7 percentage points higher, on average, p = 0.04). There was no significant effect of calcium on BAP alone at any stage (p-values: 0.61, 0.20, 0.32 for 2^nd^ trimester, 3^rd^ trimester, and 1-month postpartum, respectively)(data not shown). Adjusting for age, primigravidity, baseline dietary calcium and total energy intakes, and baseline NTx/BAP ratio, the calcium group had lower, though not statistically significant, NTx/BAP ratio estimates at the 2nd (−10.1%, p = 0.32) and 3rd trimester (−13.4%, p = 0.20) visits. By 1-month postpartum, those in the calcium group had statistically significant lower NTx/BAP ratios than those in the placebo group (−21.5%, p = 0.04) indicating a greater net reduction in bone loss in the supplement group by the end of follow-up.

The subset of women with SOS available (N = 290) were not significantly different than those who did not have the measurements available except for years in school (those with SOS had 0.6 more years, p = 0.01) and total energy intake (women with SOS consumed ~190 kcal less, on average, p < 0.001). While radial SOS decreased over the course of pregnancy in both groups, declines in the supplement group were relatively attenuated and, by 1-month postpartum, those in the supplement group had higher, though not significantly, radial SOS than those in the placebo group (p = 0.13) (data not shown). Calcium was associated with an overall average increase of 9.05 m/s in radial SOS relative to placebo though this difference was not significant (p = 0.216). However, among those subjects who consumed 50% or more of pills (N = 251), calcium was associated with an increase of 26.3 m/s in radial SOS relative to placebo by 1-month postpartum (p = 0.03). Among those subjects who consumed at least 75% of pills, calcium supplementation was associated with an increase of 59.0 m/s in radial SOS relative to placebo by 1-month postpartum (p = 0.009).

## Discussion

In this randomized controlled trial, a 1,200 mg daily calcium carbonate supplement administered during pregnancy and the early postpartum period was associated with reductions in NTx, compared to placebo, both during pregnancy and at one month postpartum, indicating that dietary calcium supplementation may help to suppress maternal bone mobilization. These effects were stronger with increasing treatment compliance, suggesting a dose–response effect, with a greater than 22% average overall reduction observed among the most compliant women. These results are consistent with a previous randomized crossover trial in a small group of women which showed that dietary calcium supplementation reduced NTx levels by an average of 14% when administered in the third trimester of pregnancy [23]. To place the magnitude and direction of these changes into context, this is consistent with a 28% reduction in urinary NTx observed after 1-month of hormone replacement therapy among women randomized to receive 0.625 mg conjugated equine estrogen (Premarin, Wyeth Ayerst, Philadelphia, PA) [41].

The results of this study are also consistent with the findings of a study among 36 pregnant Chinese women with low habitual dietary calcium intake that found calcium supplementation was associated with significant decreases in markers on bone resorption; although in contrast to our findings, they also reported increases in bone formation [25]. Unlike our study, calcium was provided by supplementing the “usual diet” with 45 g milk powder (350 mg calcium) or milk powder plus 600 mg calcium supplement (950 mg calcium). In that study, dietary calcium supplementation during pregnancy was associated, in a dose-dependent manner, with greater BMD measured by DXA at 6 weeks postpartum at the spine and whole body (p < 0.05), but not at the hip site.

In the present study, calcium was associated with significantly higher radial SOS, a marker of bone density, by 1-month postpartum among the most compliant subjects. While the overall effect, including all subjects regardless of compliance, was not statistically significant, the direction of the effect is consistent with our hypothesis and radial SOS measurements were available in only about half of the subjects, thus, the study was underpowered to detect an effect of calcium on SOS. In addition, calcium’s impact on bone density may differ depending on the type of bone. We measured SOS in the distal radius, a site with a predominance of cortical bone, and calcium may be acting on bone sites where trabecular bone dominates.

In a study of 125 Gambian women, supplementation with 1,500 mg/day calcium was associated with lower BMD measured by DXA in a subset of participants at the distal and midshaft of the radius, but with increases in measures of BMD in the lumbar spine and whole body [24]. Like the Chinese study, the Gambian study also measured the effect of calcium supplementation among women with low dietary calcium intake. However, unlike our study and the one by Liu et al. [25], the Gambian study did not continue supplementation into the postpartum period which may be partially responsible for their findings of rebound demineralization following cessation of lactation [42]. We found that the ratio of bone resorption-to-bone formation was significantly lower in the calcium group by 1-month postpartum suggesting that calcium is effective in reducing net bone loss measured after pregnancy. The observed effects at 1-month postpartum were being driven by lactating women in our study which suggests that the need for continuation of calcium supplementation may extend into the postpartum period.

A limitation of our study is that we used QUS, and not DXA, to assess bone quality in pregnant women and this measurement was available in only about half of the women. QUS has been demonstrated to predict fracture risk [43] and has been widely used in epidemiologic studies to measure bone density particularly where DXA is not available [44] or not advisable, such as during pregnancy [37–39] due to the potential for radiation exposure to the fetus. QUS has been found to be well-correlated with DXA at all sites measured over 7 years of follow-up [45] and provides our study with the advantage that we were able to include repeated measures of bone density, in addition to biochemical markers of bone turnover, over the entire course of pregnancy and the early postpartum period.

Pregnancy and lactation may impact a woman’s peak bone mass which is an important determinant of subsequent osteoporosis risk [46]. In addition, calcium may also have potential benefits for child bone health [16, 17, 47]. The possibility that intrauterine programming of fetal bone growth may be an important determinant of osteoporosis and the risk of other chronic diseases in later life is now being considered [48]. New evidence indicates that maternal dietary deficiencies during pregnancy may be associated with lower peak bone mass in offspring later in life [16, 17].

Average baseline dietary calcium intake for women in our trial was within the current recommended dietary guidelines of 1,000-1,300 mg/day for pregnant and lactating women [18]. It is possible that high amounts of calcium are needed to counterbalance the nutritional needs of the developing fetus [49]; thus, previous trials among women with low habitual dietary calcium intakes may have been unable to detect an effect. Bone mineralization does not depend solely on the availability of calcium: protein, energy, and other nutrients are also important to bone formation and mineralization. Vitamin D is essential for calcium homeostatis and now recognized as an important nutrient for bone health including modest support for maternal vitamin D status and increased offspring bone mass among [50]. However, this study was planned and carried out on the basis of the 1997 IOM guidelines [28]. Vitamin D was not specifically recommended with calcium supplementation as is currently common practice. Nonetheless, other prior studies of calcium supplementation in adult pregnancy [23–25], to which we compare our results, also did not measure or administer Vitamin D. One small randomized study of pregnant Brazilian adolescents with habitually low calcium intake [51] found that 600 mg calcium carbonate plus vitamin D_3_ (200 IU) resulted in higher lumbar spine bone mass and a reduced rate of femoral neck bone loss during lactation which is consistent with our results. The maternal response to fetal calcium demand may also be highly individualized and other genetic, hormonal, or lifestyle factors may be involved [52].

## Conclusion

In summary, dietary calcium intake likely plays a modest, but important role in suppressing maternal bone mobilization during pregnancy and the early postpartum. Calcium supplementation during pregnancy may also reduce the risk of hypertensive disorders of pregnancy [53, 54], pre-eclampsia [55, 56], and lead exposure [57] which themselves pose risks to the mother and fetus. The risks posed by calcium supplementation at levels approximating the upper limit of recommended daily intake are relatively minor [2, 18] and U.S. guidelines for calcium in pregnancy and lactation are based on studies in non-pregnant adults [2]. The World Health Organization now recognizes the importance of calcium supplementation in pregnancy [58]. Thus, dietary supplementation of calcium intake among pregnant and lactating women should be considered particularly in populations where dietary calcium intake is low.

## Abbreviations

BCE: Bone collagen equivalents
BAP: Bone-specific alkaline phosphatase
BMD: Bone mineral density
DXA: Dual-energy x-ray absorptiometry
QUS: Quantitative ultrasound
SOS: Speed of sound
NTx: Urinary cross-linked N-telopeptides of type I collagen.
